# Optical Dissection of Neural Circuits Responsible for *Drosophila* Larval Locomotion with Halorhodopsin

**DOI:** 10.1371/journal.pone.0029019

**Published:** 2011-12-28

**Authors:** Kengo Inada, Hiroshi Kohsaka, Etsuko Takasu, Teruyuki Matsunaga, Akinao Nose

**Affiliations:** 1 Department of Complexity Science and Engineering, Graduate School of Frontier Sciences, University of Tokyo, Kashiwanoha, Kashiwa, Chiba, Japan; 2 Department of Physics, Graduate School of Science, University of Tokyo, Hongo, Bunkyo-ku, Tokyo, Japan; Freie Universitaet Berlin, Germany

## Abstract

Halorhodopsin (NpHR), a light-driven microbial chloride pump, enables silencing of neuronal function with superb temporal and spatial resolution. Here, we generated a transgenic line of *Drosophila* that drives expression of NpHR under control of the Gal4/UAS system. Then, we used it to dissect the functional properties of neural circuits that regulate larval peristalsis, a continuous wave of muscular contraction from posterior to anterior segments. We first demonstrate the effectiveness of NpHR by showing that global and continuous NpHR-mediated optical inhibition of motor neurons or sensory feedback neurons induce the same behavioral responses in crawling larvae to those elicited when the function of these neurons are inhibited by Shibire^ts^, namely complete paralyses or slowed locomotion, respectively. We then applied transient and/or focused light stimuli to inhibit the activity of motor neurons in a more temporally and spatially restricted manner and studied the effects of the optical inhibition on peristalsis. When a brief light stimulus (1–10 sec) was applied to a crawling larva, the wave of muscular contraction stopped transiently but resumed from the halted position when the light was turned off. Similarly, when a focused light stimulus was applied to inhibit motor neurons in one or a few segments which were about to be activated in a dissected larva undergoing fictive locomotion, the propagation of muscular constriction paused during the light stimulus but resumed from the halted position when the inhibition (>5 sec) was removed. These results suggest that (1) Firing of motor neurons at the forefront of the wave is required for the wave to proceed to more anterior segments, and (2) The information about the phase of the wave, namely which segment is active at a given time, can be memorized in the neural circuits for several seconds.

## Introduction

A major challenge in neuroscience today is to understand neural information processing in the brain. Techniques to acutely inhibit neural activity and/or synaptic release provide effective methods towards this goal [1]–[3]. In particular, the use of halorhodopsin from the archaebacterium *Natronomonas pharaonis* (NpHR) is promising because it enables superior temporal and spatial control [4]–[5]. NpHR is a chloride pump, which, when activated by a yellow light, suppresses the firing of neurons. NpHR-mediated neuronal silencing has been demonstrated electrophysiologically *in vitro* [6] and applied *in vivo* to specific neurons in *Caenorhabditis elegans* [7], zebrafish [8]–[9] and rodents [6], [10]–[11]. To date, however, there has been no report on the use of NpHR in *Drosophila*. Although Shibire^ts^, a dominant temperature-sensitive mutation of dynamin, has been used to temporarily inhibit neural activity in *Drosophila* [1]–[2], it is difficult to make rapid changes in temperature. Therefore, control of neural activity on the time scale of tenths of seconds is impossible with this technique.

Peristaltic movement is a main behavior of *Drosophila* larvae and is generated by a rhythmic wave of muscular contraction that propagates from the posterior to anterior segments [12]–[13]. As in other animals, this movement is thought to be regulated by neural networks called central pattern generators (CPGs) that generate periodic motor outputs for rhythmic movements. While the neural basis of CPG networks has been analyzed in lamprey [14], lobster [15] and leech [16] among others, identities of the CPG neurons remain to be explored in the *Drosophila* larval network. However, the *Drosophila* larval circuits provide a promising system to apply optogenetics to the identification and characterization of the component neurons of the CPG, owing to its optical transparency, relatively simple neural structure and sophisticated genetic techniques (e.g., [17]–[20]).

A *Drosophila* larva consists of 11 body segments. The excitation of glutamatergic motor neurons in the ventral nerve cord induces contraction of the corresponding muscles in each segment [13], [21]. During the peristaltic propagation from tail to head, the contraction of muscles in one segment seamlessly propagates to the next anterior segment. Therefore, motor neurons in each segment have to be sequentially activated from the posterior to anterior segments. Indeed, previous electrophysiological recordings have revealed rhythmic bursts of activity in motor neurons that occur concurrently with locomotive waves [13], [22]–[23]. How the rhythmic activity in motor neurons is regulated by the central circuits, however, remains unknown.

In many motor circuits, sensory feedback modulates the activity of the central motor circuits to ensure that the final motor output meets the behavioral demand [24]–[25]. This is also the case for motor circuits that regulate *Drosophila* larval peristalsis [23], [6]–[27]. In each abdominal hemisegment, there are 43 sensory neurons, which are divided into three major types: external sensory (es) organs, chordotonal (cho) organs, and multidendritic (md) neurons [28]. Among these, subsets of md neurons have been shown to be particularly important for normal peristaltic locomotion [23], [27]. When the function of these neurons is temporally inhibited by Shibire^ts^, the larval peristalsis dramatically slows down, indicating that sensory feedback plays a crucial role in propagating the wave [27]. Recently, the TRP channel TRPN1/NompC has been implicated in the regulation of this sensory feedback [29].

Here we are interested in the mechanism underlying the seamless activation of motor neurons in successive segments, particularly how it is generated by the central circuits in *Drosophila* larvae. For this investigation, we generated a transgenic line that allows NpHR to be expressed under control of the GAL4/UAS system and performed temporally and spatially restricted inhibition of specific component neurons in the motor circuits. Our optogenetic analyses showed that activation of motor neurons is necessary for the wave of muscular contraction to proceed to more anterior segments. Based on our observations, a possible mechanism for information flow in the motor circuits is discussed.

## Results

### Optical inhibition of motor neurons with NpHR during larval crawling

**Figure 1:**
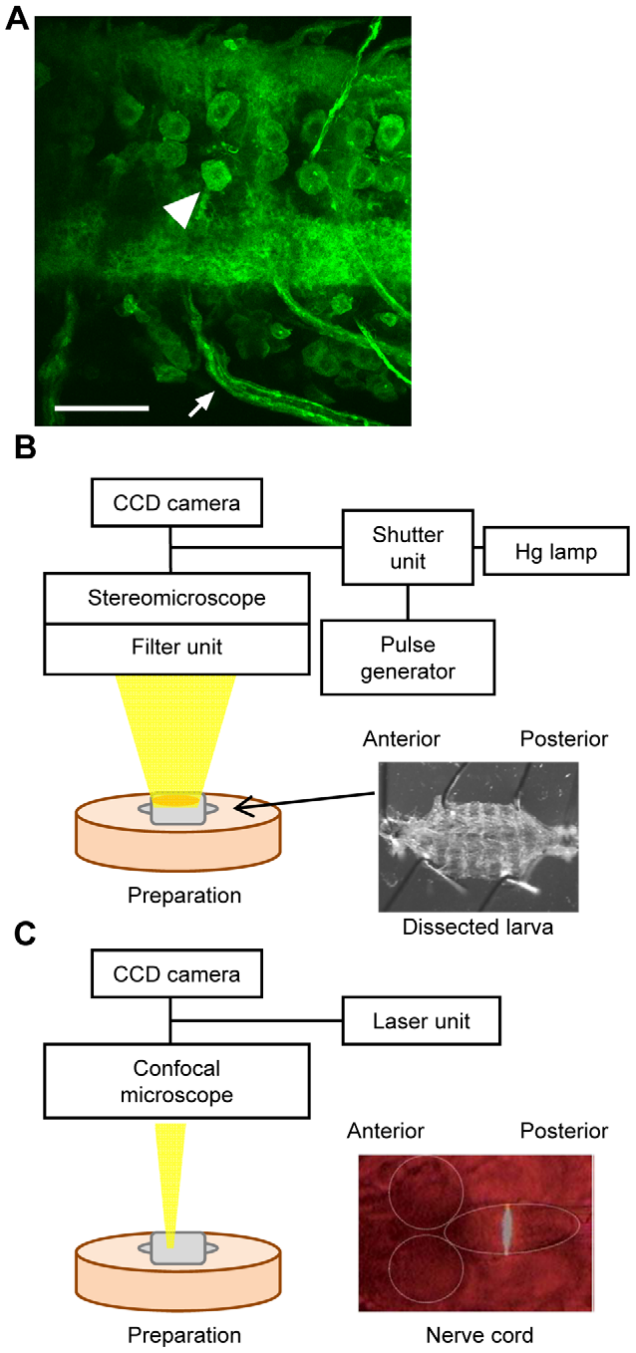
Expression of eNpHR and optical systems used for neuronal silencing. (A) Representative larval motor neurons expressing eNpHR-YFP. An arrowhead and arrow indicate a cell body and axons, respectively. *OK6-Gal4* was used to express eNpHR in motor neurons. Scale bar, 30 µm. (B) The optical system used to stimulate the entire body of a larva. Wavelength of excitation light from mercury lamp was adjusted by filter unit (excitation 540–580 nm) for eNpHR activation. The light was applied to a crawling larva or dissected larva pinned on a silicon dish (right panel). (C) The confocal system used for spatially-restricted stimulation. Laser light was applied to a restricted region in the nerve cord (right panel).

**Figure 2:**
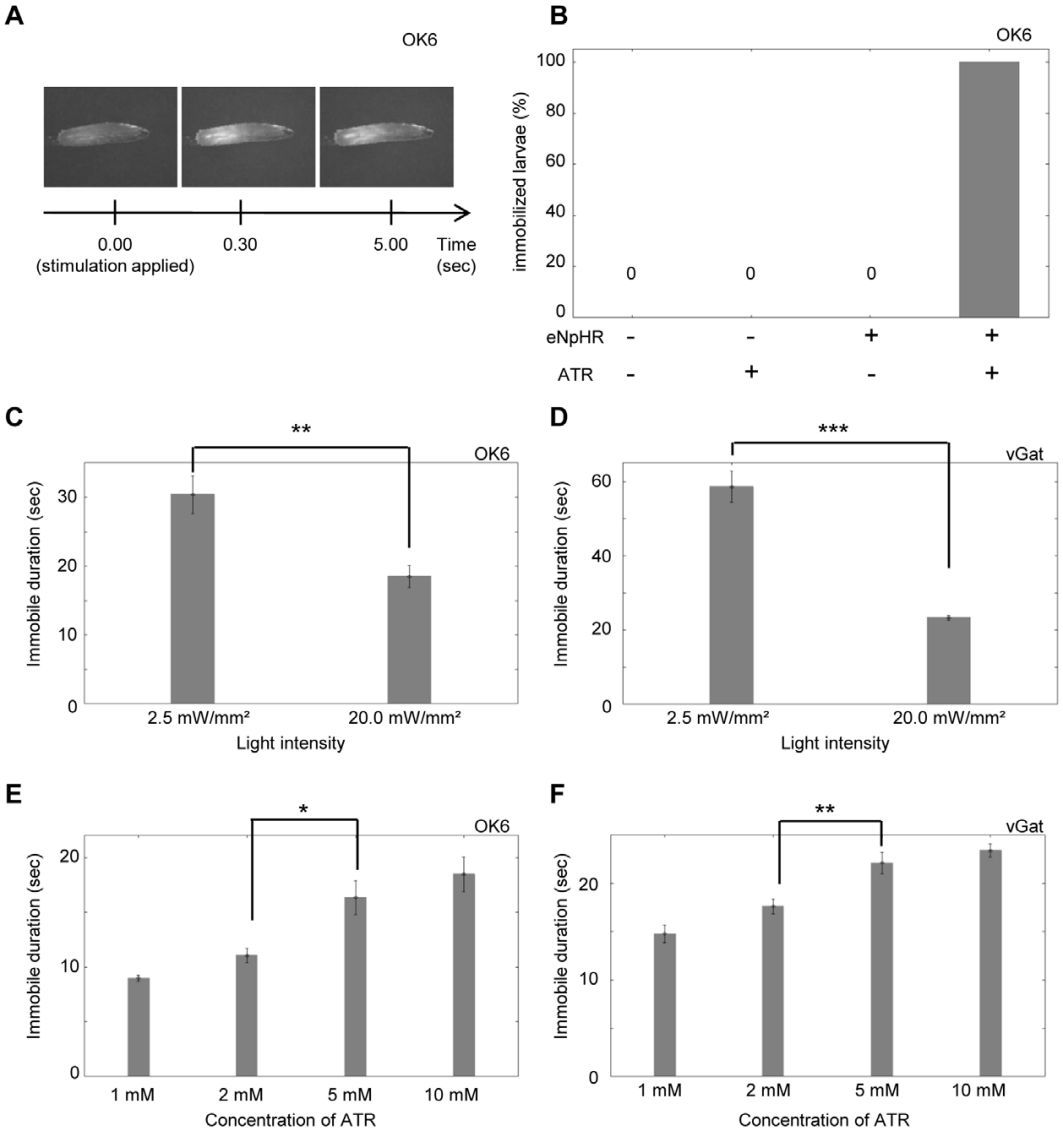
Optical inhibition of motor neurons. Optical inhibition of motor neurons immobilized the larvae. *OK6-Gal4* (A, B, C, E) and *vGat-Gal4* (D, F) were used to express eNpHR in motor neurons. (A) Postures of a larva expressing eNpHR before (left) and after light stimulation (middle, 0.5 sec; right, 5 sec). The entire body was relaxed after light stimulation. (B) Dependence on the eNpHR transgene and ATR. Percentage of larvae immobilized over 5 sec in response to the continuous optical stimulation. n = 20 for each experiment. (C–F) Dependence on light intensity (C, D) and concentration of ATR (E, F). Average duration of the effective inhibition is plotted. n = 9∼10 for each experiment. Only a single stimulation was applied in this experiment and in Figs. 3 and 4. Thus, n represents the number of stimulation as well as the number of larvae examined. Error bars represent standard error. ***p<0.001, **p<0.01, *p<0.05; Student's *t*-test (C, D), ANOVA with Tukey-Kramer post-hoc test (E, F).

We generated transgenic lines that enable expression of enhanced NpHR (eNpHR; [4]–[5]) fused with YFP (eNpHR-YFP) under the control of the Gal4-UAS system [30] and tested the effectiveness of NpHR in *Drosophila* by examining how light stimulation of neurons expressing eNpHR affects larval crawling. We first expressed eNpHR in all motor neurons and studied the effect of light stimulation. Because a chromophore of NpHR called all-*trans* retinal (ATR) is not endogenously present in *Drosophila*, unlike in mammals, the larvae were fed with ATR prior to the experiments. Confocal imaging confirmed that eNpHR was successfully expressed on the surface of cell bodies, axons and terminals of motor neurons (Fig. 1A). To activate eNpHR, a brief yellow light stimulus (several seconds) at an intensity of 20.0 mW/mm^2^ was applied under a stereomicroscope to the entire body of crawling larvae (Fig. 1B). The yellow light stimulation by itself had no effect on larval behavior. It is known that the larvae exhibit light-avoidance behavior upon stimulation with blue, violet and ultraviolet lights, but are largely unresponsive to green, yellow and red lights [31]. Since motor neurons regulate contraction of muscles, one would expect muscle relaxation upon the activation of eNpHR, as previously reported in *C. elegans* [4]. Indeed, upon light stimulation, locomotion ceased completely and all muscles relaxed instantaneously (Movie S1, Fig. 2A). This light-induced immobility was dependent on the presence of the transgenes and ATR, indicating that the light-induced immobilization is specifically due to the activation of eNpHR in motor neurons (Fig. 2B). When the light stimulation was switched off, the entire body of the larvae contracted (possibly due to post-inhibitory rebound in target neurons, see below), but later resumed normal peristaltic locomotion, indicating that the effect of optical stimulation and neuronal inactivation are reversible (Movie S1). Similar light-induced immobilization was induced when eNpHR was expressed in motor neurons with *OK6-Gal4*, *vGat-Gal4* or *C380-Gal4*, in all neurons with *actin-Gal4*, or in cholinergic neurons (including the upstream neurons of motor neurons) with *cha-Gal4* (note however that *cha-Gal4* also induces expression in subsets of motor neurons; Fig. 2 and data not shown).

We next assessed the sustained period of eNpHR-mediated inhibition. A continuous light stimulus over several tens of seconds was applied to crawling larvae expressing eNpHR. The larvae stopped their locomotion completely upon the light stimulus as described above, but resumed locomotion after several tens of seconds even in the presence of the light stimulus (Movie S2). The recovery from the inhibition is likely due to inactivation of eNpHR after a prolonged period of light stimulation. The duration of the effective inhibition (as defined by the period between the onset of optical stimulation and the first resumed movement) was dependent on the light intensity and concentration of the ATR given to the animals (Fig. 2C–F).

The inactive form of halorhodopsin can be converted to an active form by illumination with a blue light [4], [32]. We therefore investigated whether simultaneous application of yellow and blue light could elongate the effective time period of eNpHR. We applied both yellow and blue light continuously to larvae expressing eNpHR in motor neurons. Unlike when stimulated with yellow light alone, these larvae remained immobile as long as the two lights were applied (at least for 3 min, Movie S3). Thus, long-term silencing of neurons with eNpHR can be achieved by simultaneous application of yellow and blue light.

### Post-inhibitory rebound induces contraction of the larvae

**Figure 3:**
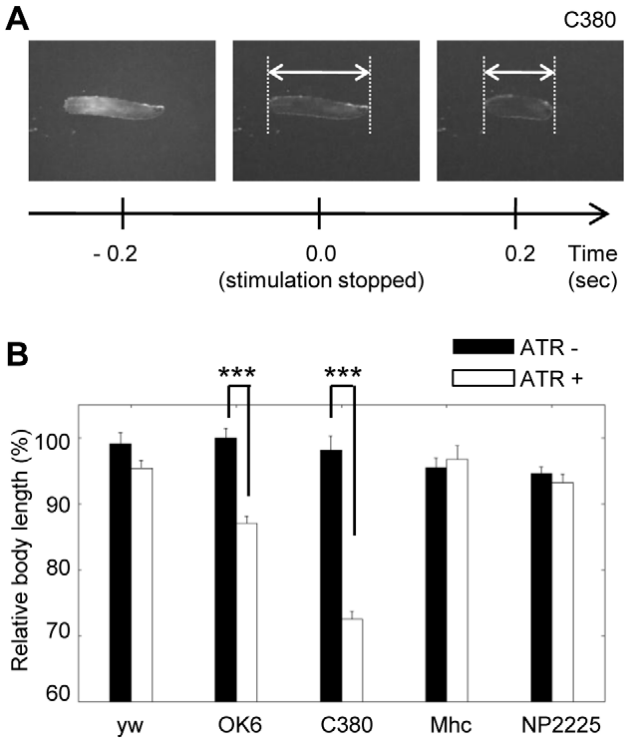
Post-inhibitory accordion-like contraction. Postinhibitory contraction of the entire larva was induced when the optical inhibition of motor neurons was switched off. (A) Postures of a larva expressing eNpHR before and after the offset of light stimulation. *C380-Gal4* was used to express eNpHR in motor neurons. (B) Postinhibitory contraction was observed when eNpHR was expressed by *OK6-Gal4* and *C380-Gal4* but not when it was expressed by *mhc-Gal4* or *NP2225-Gal4*. Relative body length, defined as the ratio of body length 0.2 sec after the offset of the light illumination to that at the time of the offset, was used as a measure. Larvae of the same genotype that were not fed with ATR were used as control. n = 7∼10. All error bars represent standard error. *p<0.05, **p<0.01; Student's *t*-test.

We found a striking behavioral response when the light was turned off after optical inhibition of neuronal populations. The offset of the illumination induced a rapid and strong contraction of the entire body of the larvae (accordion-like contraction [19], [17], Movie S1, Fig. 3A). This behavioral response is very similar to the behavior induced when all motor neurons are simultaneously activated by optical stimulation with Channelrhodopsin-2 ([17], Movie S4). It therefore seems likely that activation of motor neurons by post-inhibitory rebound induced the accordion-like contraction. Such post-inhibitory rebound has also been reported for zebrafish neurons when they are inhibited with eNpHR [8]. We quantified the accordion-like contraction by measuring the length of the larvae. The post-inhibitory accordion-like contraction was observed when eNpHR was expressed in primarily motor neurons with *OK6-Gal4*, primarily motor and sensory neurons with *C380-Gal4*, but not when eNpHR was expressed in all muscles with *Mhc-Gal4* or in sensory feedback neurons with *NP2225-Gal4* (Fig. 3B).

### Optical inhibition of sensory feedback neurons

**Figure 4:**
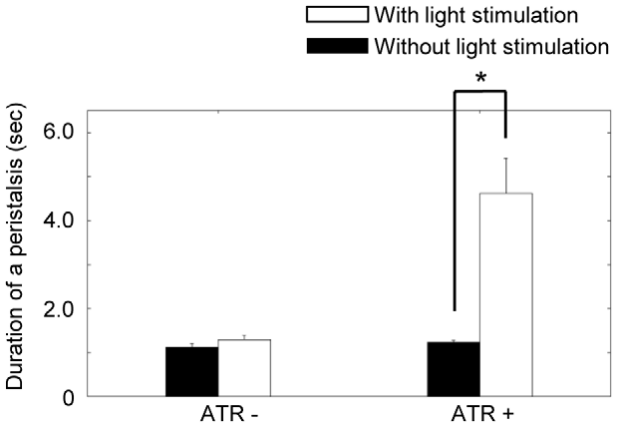
Optical inhibition of sensory neurons. Optical inhibition of sensory feedback neurons slowed down the larval crawling speed. The duration of peristalsis after 1 min of continuous stimulation with or without simultaneous application of yellow and blue lights was analyzed. *p<0.05; paired *t*-test. n = 6 for ATR^+^, n = 5 for ATR^−^.

We next examined the possibility of optically inhibiting sensory feedback neurons during larval crawling. Previous studies report that inhibition of the class I md and bipolar dendritic (bd) sensory neurons with Shibire^ts^ slows down the propagation of muscular contraction [23], [27]. We therefore studied the inactivation of these neurons with eNpHR to see whether it leads to a similar behavioral abnormality. We expressed eNpHR in class I md and bd sensory neurons with *NP2225-Gal4* and applied optical stimulation to the entire body of a crawling larva. To elongate the effective time of eNpHR, blue light was also applied as described above. The light stimulus slowed down larval crawling (Movie S5) to a similar degree as when these neurons are silenced with Shibire^ts^, suggesting that optical inhibition with eNpHR was successful. To quantify the results, we examined the wave duration (defined as the time required for a wave of muscular contraction) before and after light stimulation and in various control conditions. The duration was significantly increased by continuous optical stimulation (Fig. 4; 1.23±0.055 sec before stimulation versus 4.62±0.80 sec after stimulation; mean ± standard error; n = 6; p<0.05, paired *t*-test). The increase was not observed in larvae that were not fed ATR (Fig. 4; 1.17±0.093 sec before stimulation versus 1.29±0.11 sec after stimulation; mean ± standard error; n = 5; p>0.05, paired *t*-test) or in the larvae without the transgene (data not shown). Thus, optical inhibition with eNpHR can be used to study the function of various component neurons involved in larval locomotion.

### Probing the motor circuits with temporally restricted light stimuli

**Figure 5:**
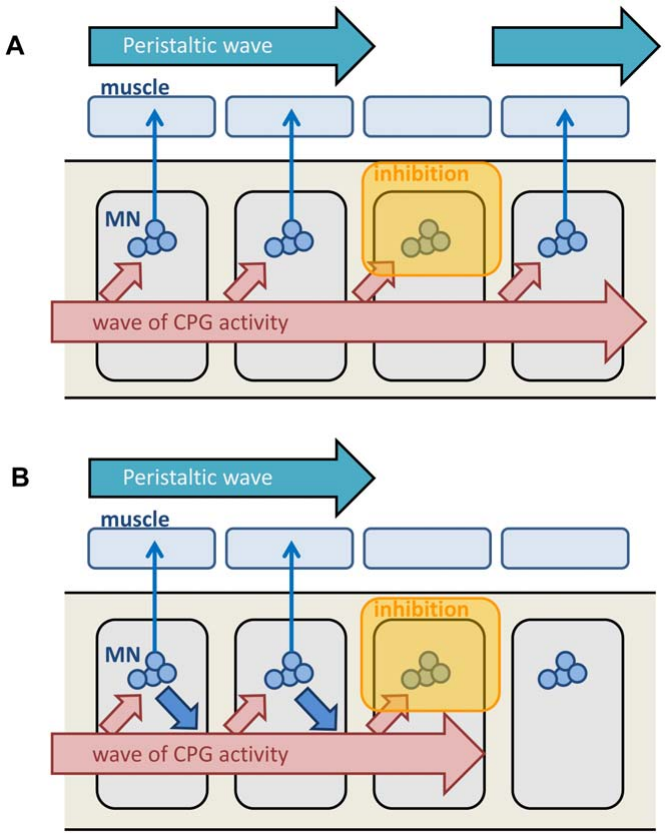
Models of activity propagation in the motor circuits. Two models of activity propagation in the motor circuits. (A) Activity propagation of the upstream central pattern generator (CPG) proceeds independent of the activity of motor neurons. (B) Activity propagation of the CPG depends on the activity of motor neurons. The results of the optical inhibition of motor neurons support this model.

We next applied a brief light stimulus (∼1 sec) to try to inhibit motor neurons in a more temporally restricted manner. The seamless propagation of muscular contraction from the posterior to anterior segments takes ∼1 second per wave in larval peristalsis. We asked what happens to the propagation when all motor neurons are transiently inhibited in the middle of the propagation. We asked this question to distinguish two alternative models of the motor circuits (see Fig. 5). On the one hand, a wave of activity may be generated by central circuits that are independent of the activity of motor neurons. On the other hand, the motor neurons may be part of the central circuits that generate the wave. If the former is the case, the activity wave within the central circuits should proceed to more anterior segments, even if the activity of motor neurons is inhibited. Thus, after transient inhibition of motor neurons, the wave of muscular constriction would reappear in a more anterior segment. If the latter is the case, the wave may be temporarily halted upon optical inhibition of motor neurons but may resume after the inhibition.

**Figure 6:**
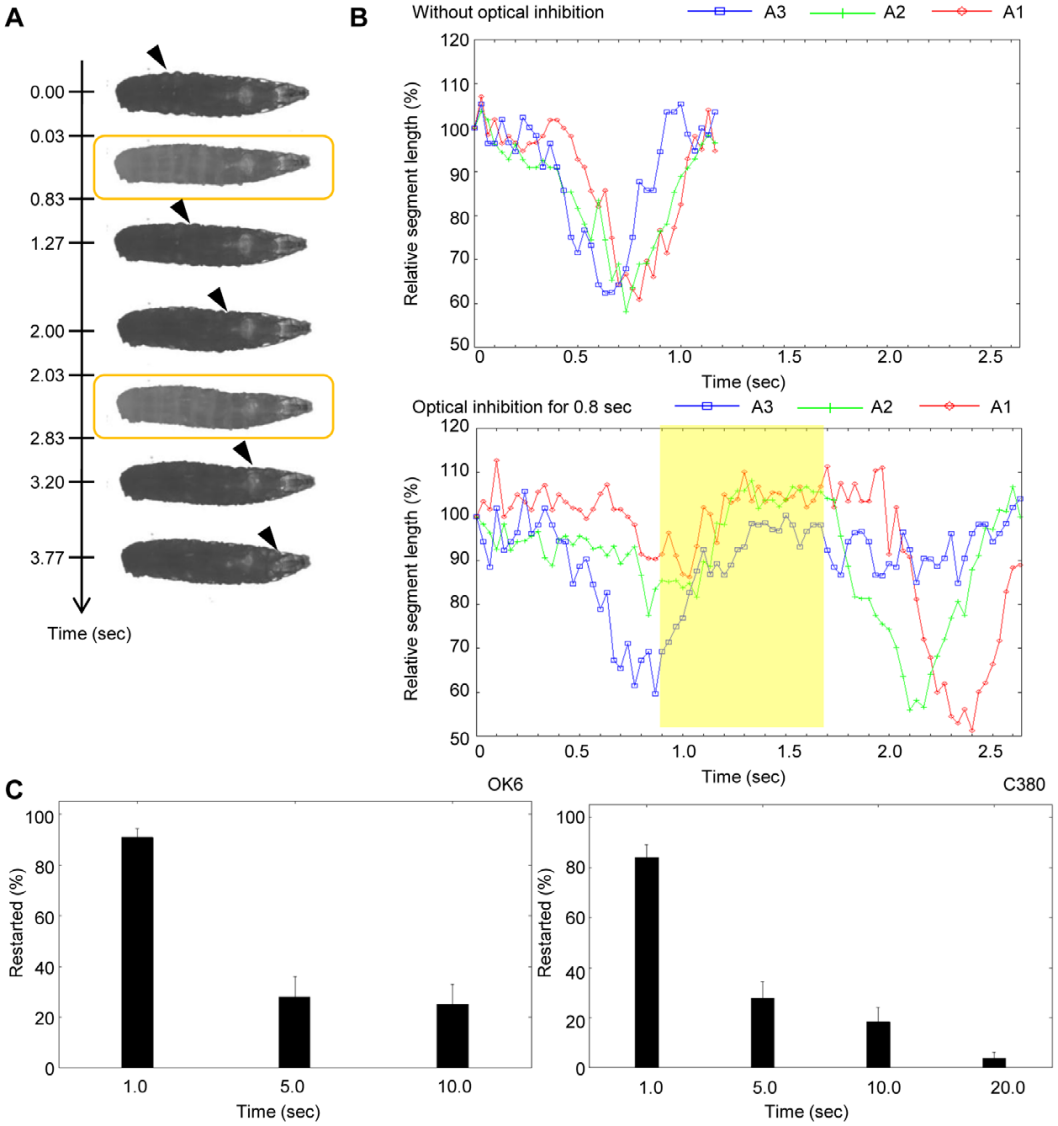
Transient motor inhibition in the crawling larvae. (A, B) Pulsed stimulation of a crawling larva with eNpHR driven by *vGat-Gal4*. (A) Postures of the larva at the time indicated. Yellow squares indicate pulsed stimulations (duration 0.8 sec). Propagation of muscular contraction was transiently stopped by the optical inhibition but resumed after the removal of the stimulation. Arrowheads indicate the contracted segments. (B) Contraction of three segments (A1–A3) with (bottom) or without (top) optical inhibition. Length of each segment normalized by the length before launch of crawling is plotted (A1, red; A2, green; A3, blue). *Top*, A3 contraction and subsequent A2 and A1 contractions were observed seamlessly in the absence of optical stimulation. *Bottom*, the optical inhibition (duration 0.8 sec) was applied after A3 contraction (yellow square). During the optical inhibition, all segments were relaxed. After the offset of the inhibition, A2 and A1contracted successively, indicating that the propagation was restarted from the segment at which propagation was inhibited. (C) Restart of peristalsis after transient optical inhibition of motor neurons in dissected larvae with *OK6-Gal4* (*left*) and *C380-Gal4* (*right*). Percentages of restart after various duration of optical inhibition are plotted. n = 14∼17 except in *C380-Gal4* duration 20.0 sec (n = 12; n represents the number of larvae examined). Error bars indicate the standard error.

We used *vGat-Gal4* to drive the expression of eNpHR in motor neurons and applied a brief light stimulus (0.8 sec) to the larvae during peristalsis. During the light stimulus, the entire body relaxed as described above, leading to the disappearance of the propagation of muscular contraction. However, the propagation reappeared when the light was turned off, at around the segment that was about to contract when the light stimulus was given. For example, in Fig. 6A, light stimuli were given twice, at around the time when segment A5 and segment A2 were about to contract (see also Movie S6). Upon each stimulus, the muscular contraction resumed at the point of disturbance after the optical inhibition was removed. To look more closely at the propagation of muscle contraction, we measured the length of each segment before, during and after the optical perturbation. An example is shown in Fig. 6B, where we applied optical perturbation after the contraction of A3. In the absence of optical perturbation, contraction of A2 and A1 was observed immediately after A3 contraction, reflecting the seamless propagation of muscular contraction (Fig. 6B, top). When the 0.8-sec optical inhibition was applied just following A3 contraction, A2 and A1 remained relaxed during optical inhibition but contracted immediately after the offset of the illumination (Fig. 6B, bottom). Such temporal pause and restart were observed upon 0.8-sec optical stimulation in 85.2% of the cases (n = 27 stimulations in 11 larvae). Thus, the propagation of muscular contraction pauses during the optical inhibition of motor neurons but can resume from the original position after the inhibition.

Because *vGat-Gal4* drives expression in GABAergic neurons in addition to motor neurons, the involvement of GABAergic neurons cannot be excluded in the above experiments. We therefore wanted to use other Gal4 lines such as *OK6-Gal4* and *C380-Gal4* to replicate the results described above. However, because inhibition of motor neurons with these Gal4 drivers induces strong post-inhibitory contraction as described above, the restart of the propagation could not be analyzed. We therefore studied the effect of the optical inhibition on fictive locomotion of larvae that are dissected and pinned down on a silicon dish ([12], Fig. 1B). Pinning of the body wall prevents the shortening of the entire musculature but allows local constriction of individual muscles, thus minimizing the effects of post-inhibitory larval shrinkage. We applied transient optical inhibition (1 sec) to dissected larvae of *vGat-Gal4*/*UAS-eNpHR* and observed the same restart of the propagation after the optical inhibition. Restart of the propagation was also seen when *OK6-Gal4* and *C380-Gal4* were used to express eNpHR in motor neurons (and in sensory neurons in the case of *C380-Gal4*) (Fig. 6C). Thus, transient inhibition of motor neurons led to a pause in the propagation of muscular contraction that resumed upon the removal of the inhibition.

The fact that the propagation resumed at the original position suggests that information about the phase of the propagation (i.e., which segment was about to contract before the light stimulus) was retained in the neural network during optical inhibition, and then read out afterwards. How long can this information be retained? To address this question, we applied light stimulation of 1.0, 5.0, 10.0 and 20.0 sec to larvae expressing eNpHR in motor neurons (Fig. 6C). When a 1.0-sec light stimulation was given, the propagation resumed in 80–90% of the cases (90.7±3.65% for *OK6-Gal4* [n = 15] and 84.0±5.01% for *C380-Gal4* [n = 15]; mean ± standard error). The rate of restart of the propagation dramatically dropped when longer light stimuli were applied. However, significant rates of successful resuming (∼20%) were still observed even when 5- or 10-sec stimuli were given (*OK6-Gal4*: 27.9±8.06% for duration 5.0 sec [n = 14], 25.0±8.03% for duration 10.0 sec [n = 14]; *C380-Gal4*: 27.7±6.62% for duration 5.0 sec [n = 17], 18.2±5.81% for duration 10.0 sec [n = 16], 3.75±2.44% for duration 20.0 sec [n = 12]). Thus, it appears that the information about the phase of the wave can be retained for more than ten seconds in the circuits.

### Spatially and temporally restricted inhibition of motor neurons revealed roles of motor neurons in activity propagation

Because larval locomotion is a successive propagation of segmental contraction, motor neurons in only a few segments are active at a given time during peristalsis. It is therefore likely that in the experiments described above, the brief light stimulus, although applied to the entire body, mostly affected the motor neurons in the few segments that were active at the time of stimulation. If so, this would suggest that the pause in the peristaltic wave is caused by temporary inhibition of the motor neurons at the forefront of the wave. To address this issue, we next applied brief light stimuli to restricted regions in the nerve cord (Fig. 1C) and asked if the optical inhibition of motor neurons in the forefront of the wave leads to the same transient inhibition and resuming of the peristalsis.

**Figure 7:**
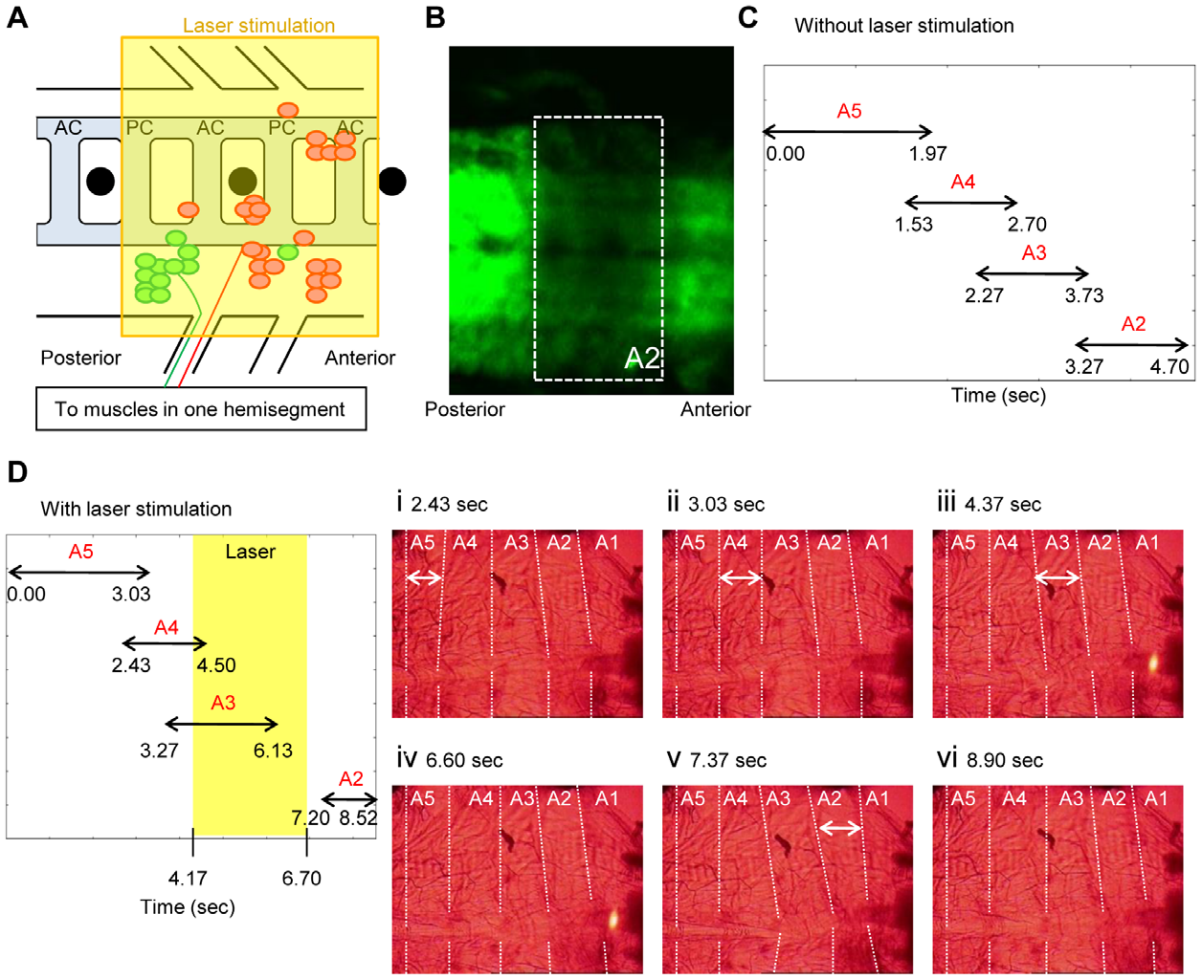
Focal inhibition of motor neurons in dissected larvae. (A) Position of the cell bodies of motor neurons that project intersegmentally (red) and segmentally (green) to muscles in a hemisegment (after [33]). Black circles represent dorsoventral channels. By applying light stimuli to the area indicated as a yellow square, motor neurons that innervate a single segment were optically silenced. (B) Photobleaching of eNpHR-YFP in the region of optical inhibition. (C) Seamless contractions of A2–5 segments in the absence of optical inhibition. Arrowed lines indicate the duration of a contraction in each segment. (D) Contraction of A2–5 when propagation was disturbed by the optical inhibition of motor neurons in A2. Arrowed lines are as in (C). A3–5 segments exhibited seamless contractions (i–iii), but A2 contraction was not observed until the stimulation was removed (iv–vi).

Using standard confocal microscopy, we applied laser stimulation to a region of the ventral nerve cord which includes all motor neurons that project to a single segment of the body wall (Fig. 7A). The region corresponds roughly to two segments in the CNS because motor neurons send their axons both segmentally (to the same segment in the body wall) and inter-segmentally (to the next posterior segment in the body wall; [33]). The location of the light illumination was determined before the light stimulus by referring to the position of the peripheral nerve and the dorsoventral channel, and was further confirmed after the stimulation by photobleaching of eNpHR-YFP in the region of optical inhibition (Fig. 7B). We found that the brief and spatially restricted laser application resulted in the same temporal cessation of the propagation as was seen when transient optical inhibition was applied to the entire body of the larvae. A typical example is shown in Fig. 7D, in which laser stimulation was applied to motor neurons innervating A2, when muscular contraction reached A3 and was about to propagate to A2 (compare with the normal peristalsis shown in Fig. 7C, see also Movie S7). The light stimulation led to relaxation of muscles in A2 while having little influence on the muscles in neighboring segments, consistent with specific inhibition of motor neurons innervating A2. The wave of muscular contraction stopped during the period of light stimulation. However, when the optical stimulation was removed, the muscular contraction restarted at A2 and propagated to the more anterior segments. The temporal cessation and restart of the muscular wave were seen when *C380-Gal4* or *OK6-Gal4* was used to express eNpHR in motor neurons (76.2% of 42 stimulations in 10 larvae for *C380-Gal4* and 91.7% of 12 stimulations in 3 larvae for *OK6-Gal4*).

Importantly, the laser stimulation alone did not initiate a wave of muscular contraction when applied to larvae in a quiescent state (between fictive locomotion, Movie S8). Thus, the restart of the propagation was not due to *de novo* generation of a wave, for example, by post-inhibitory activation of motor neurons. Another important point is that optical inhibition has to be applied at the forefront of the wave to stop the propagation; light stimulation at a more posterior or anterior segment had no effect (Movie S9). Again, this argues against the possibility that the restart of the wave is generated by post-inhibitory rebound. These results indicate that firing of motor neurons at the forefront of the wave is necessary for the peristalsis to proceed to more anterior segments.

## Discussion

### Optical inhibition of neuronal activity in *Drosophila* with halorhodopsin

Inhibition of neural activity is an effective method for dissecting the function of a neural network. In this study, we demonstrated successful use of halorhodopsin for temporally and spatially restricted neuronal silencing in *Drosophila*. Shibire^ts^ has been used in conjunction with the Gal4-UAS system to temporally inhibit the function of specific neurons in various *Drosophila* neural circuits [1]–[2], [34]–[38]. However, because it relies on changes in temperature, Shibire^ts^-mediated inhibition is not suitable for analyses of information processing in neural circuits that operate on the order of milliseconds or tenths of seconds. Furthermore, because the expression of Gal4 lines is rarely restricted to specific cell types or neuronal regions, it is usually difficult to target neuronal inhibition to specific cell populations in the nervous system (e.g., motor neurons in one segment). In contrast, light-controllable halorhodopsin provides a superior spatiotemporal resolution and promises to advance the dissection of neural circuits in *Drosophila*.

We first demonstrated the utility of NpHR in *Drosophila* by expressing it in motor neurons or sensory feedback neurons and examining the effect of global light illumination on larval crawling. The optical inhibition of motor neurons induced complete paralysis, whereas that of sensory feedback neurons merely slowed down the peristalsis. These behavioral responses are similar to those elicited when the function of these neurons is inhibited with Shibire^ts^. For example, previous studies reported that inhibition of feedback neurons with Shibire^ts^ decreased the speed of larval locomotion ∼4 fold [23], [27]. Inhibition of these neurons with NpHR also resulted in ∼4 fold decrease in the speed of larval locomotion (note, however, that these experiments were done at different temperatures and thus direct comparison of the actual speed is not possible).These results indicate that eNpHR efficiently inhibits neural function in *Drosophila*. It should be noted, however, that multiple copies of eNpHR are often required to obtain a maximum level of neural inhibition. Thus, this approach may not be utilized for experiments in which introduction of multiple copies of eNpHR is difficult.

We then inhibited neuronal function in a more temporally and/or spatially restricted manner, taking advantage of NpHR. When we transiently (∼0.8 sec) inhibited motor neurons by application of a pulsed light to the entire body, the larval peristalsis paused during inhibition but resumed after the offset of the inhibition. We also succeeded in optical inhibition of motor neurons in a specific region at a specific time during fictive locomotion of dissected larvae and showed that activation of motor neurons at the forefront of the wave is critical for propagation of the wave. These results demonstrate that NpHR provides a powerful tool for dissecting the spatio-temporal dynamics of neural circuits in *Drosophila*.

### The generation of activity propagation in the central circuits

In larval locomotion, the spatiotemporal pattern of motor activation is thought to be generated by CPG circuits within the CNS. Since the final output of the central circuits is the successive activation of motor neurons in neighboring segments, the activity of the CPG itself must also be coordinated between segments [24]. The fact that the propagation of muscular contraction is halted by temporal inhibition of motor neurons but resumes after the inhibition points to two important features of the circuits that generate the motor pattern.

First, activity of motor neurons is required for activity propagation within the central circuits. If the upstream CPG generated a wave of activity independent of the firing of motor neurons, neurons in more anterior segments would fire at the appropriate time, even if the activity of motor neurons in one or a few segments are inhibited. Thus, the wave of muscular contraction would proceed, skipping the segments that are directly inhibited by optic silencing (Fig. 5A). Instead, we observed that the propagation is temporarily stopped when the activity of motor neurons in one or a few segments is inhibited. The results suggest that motor neurons are part of the circuit that generates the wave: motor neurons not only receive output drive from the central circuits but also contribute to the activity propagation within the circuits (Fig. 5B). How motor neurons contribute to the generation of the wave remains to be investigated. One possibility is that motor neurons not only send information to muscles but also to interneurons in the CPGs as seen in other systems (e.g. collateral axon projection of motor neurons that innervate Renshaw cells in mammals). No anatomical evidence for the presence of axon collaterals of motor axons has been reported; however, we observed that the presynaptic marker Synaptotagmin-GFP localizes not only in the terminals on muscles but also in bouton-like structures in the CNS, when it is expressed in motor neurons (Y. Itakura, H.K. and A.N., unpublished data). Thus, it is possible that motor neuron neurites are outputting information as well as receiving information. Another possibility is that muscle contraction driven by motor neurons and the resultant sensory feedback contribute to the propagation. However, we think this possibility is unlikely because the peristaltic wave can occur even in the absence of sensory feedback; although inhibition of sensory feedback slows down the speed of the wave, it does not stop the wave [12], [27]. The activity propagation can therefore be generated autonomously within the central circuits. Thus, we suggest that direct information flow from motor neurons to interneurons is responsible for the wave propagation.

Another feature of the circuits that was revealed in our experiments is that information about the temporal order of segmental contractions, namely which segment is active at a given time, can be retained in the neural circuits. When the inhibition of motor neurons was ceased, the wave resumed at the position that was about to contract when the inhibition was applied. This was observed when the inhibition was applied to all motor neurons in intact or dissected larvae and when the inhibition was applied to motor neurons at the forefront of the wave in dissected larvae. The observation indicates some sort of memory system in the circuits. Existence of the memory of the phase of locomotion has also been suggested in other systems. For example, during fictive locomotion in vertebrates, rhythmic motor activity can reappear after cycles of skipped locomotion, maintaining the phase of the original cycle [39]. Our experiments suggest that the memory can last over ten seconds, which is much longer than the period required for a wave of peristalsis (∼1 sec). How the memory is retained in the circuits remains to be investigated. One possibility is that some gate-keeping interneurons continue to fire during the optical inhibition of motor neurons. For example, combined action of the gate-keeping interneurons and motor neurons may be required for the wave to proceed. It is important to note that post-inhibitory activation of motor neurons alone does not initiate a wave. Focal optical inhibition of motor neurons in a resting state or in segments other than those at the forefront of the wave does not induce *de-novo* generation of the wave. Furthermore, even when optical inhibition was globally applied to all motor neurons in intact or dissected larvae, the restart of the wave only occurred at the forefront of the wave but not in other segments. Thus, it is likely that memory retained in the central circuits together with the release of the motor neuron inhibition drives the re-initiation of the wave.

In summary, we have shown here the successful use of eNpHR for the dissection of neural circuits in *Drosophila*. The superior temporal and spatial resolution of eNpHR enabled precise neuronal silencing that is not possible with previously available tools such as Shibire^ts^. Initial application of this tool to the larval motor circuits revealed two important features of the circuits: involvement of the motor neuron activity in wave propagation and the existence of a memory retention system in the circuits. Future identification of interneuronal populations in the central circuits will allow more detailed analyses of the cellular underpinnings of these features. eNpHR-mediated neuronal silencing may also be combined with electrophysiology or calcium imaging [2]–[3] to allow more detailed dissection of the functional characteristics of the circuits.

## Materials and Methods

### Fly stocks

All fly stocks were reared at 25°C. The following gal4 lines were used to express eNpHR in specific cells: *actin-Gal4* (all cells; [40]), *cha-Gal4* (cholinergic neurons; [41]), *NP2225-Gal4* (bd and multidendritic class I sensory neurons; [27], [42]), *mhc-Gal4* (muscles; [43]), *OK6-Gal4* (motor neurons; [44]), *C380-Gal4* (motor neurons and sensory neurons; [44]), and *vGat-Gal4* (most if not all of motor neurons and a small subsets of GABAergic neurons; [45] and our unpublished observation). We generated 26 UAS lines carrying enhanced NpHR2.0 (eNpHR hereafter; [4]–[5]). To facilitate visualization of the cells expressing eNpHR, the sequence of eNpHR was followed by yellow fluorescent protein (YFP). From the 26 *UAS-eNpHR-YFP* lines, three insertions with strong expression, one on chromosome II (*UAS-eNpHR-50C*) and the other two on chromosome III (*UAS-eNpHR-19C* and *UAS-eNpHR-34B*), were chosen for the analyses. We found that in general, multiple copies of UAS-eNpHR were required to obtain effective inhibition with eNpHR. We therefore generated a line homozygous for the three *UAS-eNpHR* insertions (*UAS-eNpHR-50C*; *UAS-eNpHR-19C*, *UAS-eNpHR-34B*). This line was utilized for expression of eNpHR with *actin-Gal4*, *cha-Gal4*, *NP2225-Gal4*, *mhc-Gal4*, *C380-Gal4*, and *vGat-Gal4*. For expression with *OK6-Gal4*, we generated a line homozygous for *OK6-Gal4* (on chromosome II) and a UAS insertion (*UAS-eNpHR-19C* on chromosome III). The *UAS-shibire^ts^* [1] line was used to express Shibire^ts^ in specific tissues and *y*, *w* was used as a control.

### Optical stimulation

All experiments were performed at 25°C. 1^st^ instar larvae expressing eNpHR were raised in the dark on an apple-juice agar plate covered with yeast paste containing ATR until they were utilized for experiments at the third instar larval stage. The concentration of ATR was 10 mM unless otherwise mentioned.

#### Optical stimulation of crawling larvae

Before stimulation, a larva was rinsed briefly to remove residual yeast paste and transferred to a new plate. The larva was allowed to move freely for several seconds before the analyses to adjust to the new environment. Optical stimulation was performed using a stereomicroscope (SZX16, Olympus), mercury lamp (U-RFL-T, Olympus), and a filter unit (SZX2-FRFP2, Olympus), as depicted in Fig. 1b. The wavelength of excitation from the mercury lamp was adjusted by a filter unit for optimal eNpHR activation (excitation 540–580 nm). A light intensity of 20.0 mW/mm^2^ was used unless otherwise noted. This intensity is reported to be strong enough to activate NpHR completely in culture [7]. Power meter (Mobiken, Laser power meter, LP1, Sanwa) was used to measure light intensities. For simultaneous stimulation with a yellow and a blue light, a 470 nm blue LED (M470L1, Thorlabs) was also used. The blue illumination was applied from the side of the stereomicroscope. For the transient perturbation assay, we used a shutter unit (UNIBLITZ, Olympus) to generate pulsed optical stimulation. The switching of the shutter was regulated by a pulse generator (SEN-3301, Nihon Kohden). Videos were captured using a cooled CCD camera (XLD-V60, SONY) mounted on the stereoscopic microscope. Video data were saved as audio video interleave (avi) files by VFS42 software (version 4.01, Chori-imaging). The capture rate was 30 frames/sec.

#### Spatially restricted optical stimulation

Larvae were dissected and pinned down on a silicon-coated dish in 2 mM Ca^2+^ Ringer's solution (in mM: 130 NaCl, 5 KCl, 2 CaCl_2_, 2 MgCl_2_, 36 sucrose, and 5 HEPES [pH 7.3]) as described previously [46]. Spatially restricted optical stimulations were applied using an FV1000 confocal microscope (Olympus) under a 10× objective lens (NA 0.40) as depicted in Fig. 1C. We used a 543-nm laser with 100% intensity for eNpHR stimulation and a 515-nm laser with 100% intensity for photobleaching. The procedures of optical stimulation and subsequent photobleaching were controlled by Fluoview software (FV10-ASW, Olympus). Videos were captured by a cooled CCD camera (ExwaveHAD, SONY) mounted on the confocal microscope as described above.

### Data analysis

The avi-style video files were analyzed with ImageJ (version 1.42q). The length of the body and segments were measured manually by a straight-line selection tool in ImageJ. The larval body was divided into several parts and summation of the length of these parts was defined as body length if the larval body was crooked. Immobile duration was defined as the time between the onset of optical stimulation and the first movement of larvae, which is typically a twitch of mouth hock. Duration of larval crawling was determined by measuring the time required for 3–5 continuous propagations and by dividing the time by the number of propagations. All statistical analyses were performed using Microsoft Excel 2007 (Microsoft) and JMP (version 9.0.2, SAS Institute).

## Supporting Information

Movie S1 Light-induced immobility and postinhibitory contraction. (OK6-Gal4;UAS-eNpHR).
(WMV)

Movie S2 Resumed locomotion under stimulation. (OK6-Gal4 was used to express eNpHR).
(WMV)

Movie S3 Prolonged immobility induced by blue and yellow lights. (OK6 was used to express eNpHR). After 2 min stimulation. After 5 min stimulation.
(WMV)

Movie S4 Accordion-like contraction induced by optical stimulation with ChR2. (OK6 was used to express H134R-ChR2).
(WMV)

Movie S5 Optical inhibition of sensory feedback neurons. (NP2225-Gal4 was used to express eNpHR). Before inhibition. After 1 min inhibition.
(WMV)

Movie S6 Application of brief light stimuli (0.8 sec). (vGat-Gal4 was used to express eNpHR).
(WMV)

Movie S7 Laser inhibition of motor neurons in A2. Half speed. (C380 was used to express eNpHR).
(WMV)

Movie S8 Laser stimulation in a quiescent state. (C380 was used to express eNpHR).
(WMV)

Movie S9 Silencing motor neurons in posterior segments when anterior segments contract. (C380-Gal4×UAS-eNpHR).
(WMV)

## References

[1] Kitamoto T (2001). Conditional modification of behavior in *Drosophila* by targeted expression of a temperature-sensitive shibire allele in defined neurons.. J Neurobiol.

[2] Luo L, Callaway EM, Svoboda K (2008). Genetic dissection of neural circuits.. Neuron.

[3] Deisseroth K (2011). Optogenetics.. Nat Methods.

[4] Gradinaru V, Thompson KR, Deisseroth K (2008). eNpHR: a Natronomonas halorhodopsin enhanced for optogenetic applications.. Brain Cell Biology.

[5] Gradinaru V, Zhang F, Ramakrishnan C, Mattis J, Prakash R (2010). Molecular and cellular approaches for diversifying and extending optogenetics.. Cell.

[6] Zhao S, Cunha C, Zhang F, Liu Q, Gloss B (2008). Improved expression of halorhodopsin for light-induced silencing of neuronal activity.. Brain Cell Biology.

[7] Zhang F, Wang L -P, Brauner M, Liewald JF, Kay K (2007b). Multimodal fast optical interrogation of neural circuitry.. Nature.

[8] Arrenberg AB, Bene FD, Baier H (2009). Optical control of zebrafish behavior with halorhodopsin.. PNAS.

[9] Schoonheim PJ, Arrenberg AB, Del Bene F, Baier H (2010). Optogenetic localization and genetic perturbation of saccade-generating neurons in zebrafish.. J Neuroscience.

[10] Gradinaru V, Mogri M, Thompson KR, Henderson JM, Deisseroth K (2009). Optical deconstruction of parkinsonian neural circuitry.. Science.

[11] Tønnesen J, Sørensen AT, Deisseroth K, Lundberg C, Kokaia M (2009). Optogenetic control of epileptiform activity.. PNAS.

[12] Fox LE, Soll DR, Wu C-F (2006). Coordination and modulation of locomotion pattern generators in *Drosophila* larvae: Effects of altered biogenic amine levels by the tyramine-hydroxlyase mutation.. J Neuroscience.

[13] Peron S, Zordan MA, Magnabosco A, Reggiani C, Megighian A (2009). From action potential to contraction: Neural control and excitation-contraction coupling in larval muscles of *Drosophila*.. Comparative Biochemistry and Physiology, Part A.

[14] Grillner S (2006). Biological pattern generation: the cellular and computational logic of networks in motion.. Neuron.

[15] Marder E, Bucher D (2007). Understanding circuit dynamics using the stomatogastric nervous system of lobsters and crabs.. Annu Rev Physiol.

[16] Friesen WO, Kristan WB (2007). Leech locomotion: swimming, crawling, and decisions.. Curr Opin Neurobiol.

[17] Schroll C, Riemensperger T, Bucher D, Ehmer J, Völler T (2006). Light-induced activation of distinct modulatory neurons triggers appetitive or aversive learning in *Drosophila* larvae.. Curr Biol.

[18] Zhang W, Ge W, Wang Z (2007). A toolbox for light control of *Drosophila* behaviors through Channelrhodopsin 2-mediated photoactivation of targeted neurons.. Eur J Neurosci.

[19] Hwang RY, Zhong L, Xu Y, Johnson T, Zhang F (2007). Nociceptive neurons protect *Drosophila* larvae from parasitoid wasps.. Current Biology.

[20] Pulver SR, Pashkovski SL, Hornstein NJ, Garrity PA, Griffith LC (2009). Temporal dynamics of neuronal activation by Channelrhodopsin-2 and TRPA1 determine behavioral output in *Drosophila* larvae.. J Neurophysiol.

[21] Jan LY, Jan YN (1976). L-glutamate as an excitatory transmitter at the *Drosophila* larval neuromuscular junction.. J Physiology.

[22] Cattaert D, Birman S (2001). Blockage of the central generator of locomotor rhythm by noncompetitive NMDA receptor antagonists in *Drosophila* larvae.. J Neurobiology.

[23] Song W, Onishi M, Jan LY, Jan YN (2007). Peripheral multidendritic sensory neurons are necessary for rhythmic locomotion behavior in *Drosophila* larvae.. PNAS.

[24] Friesen WO, Cang J (2001). Sensory and central mechanisms control intersegmental coordination.. Curr Opin Neurobiol.

[25] Rossignol S, Dubuc R, Gossard JP (2006). Dynamic sensorimotor interactions in locomotion.. Physiol Rev.

[26] Suster ML, Bate M (2002). Embryonic assembly of a central pattern generator without sensory input.. Nature.

[27] Hughes CL, Thomas JB (2007). A sensory feedback circuit coordinates muscle activity in *Drosophila*.. Molecular and Cellular Neuroscience.

[28] Jan YN, Jan LY, Bate M, Arias AM (1993). The peripheral nervous system.. The development of the *Drosophila* melanogaster volume II.

[29] Cheng LE, Song W, Looger L, Jan LY, Jan YN (2010). The role of the TRP channel NompC in *Drosophila* larval and adult locomotion.. Neuron.

[30] Brand AH, Perrimon N (1993). Targeted gene expression as a means of altering cell fates and generating dominant phenotypes.. Development.

[31] Xiang Y, Yuan Q, Vogt N, Looger LL, Jan LY (2010). Light-avoidance-mediating photoreceptors tile the *Drosophila* larval body wall.. Nature.

[32] Hegemann P, Oesterbelt D, Steiner M (1985). The photocycle of the chloride pump halorhodopsin. I: Azide-catalyzed deprotonation of the chromophore is a side reaction of photocycle intermediates inactivating the pump.. Embo J.

[33] Sink H, Whitington PM (1991). Location and connectivity of abdominal motoneurons in the embryo and larva of *Drosophila melanogaster*.. J Neurobiology.

[34] Waddell S, Armstrong JD, Kitamoto T, Kaiser K, Quinn WG (2000). The amnesiac gene product is expressed in two neurons in the *Drosophila* brain that are critical for memory.. Cell.

[35] Dubnau J, Grady L, Kitamoto T, Tully T (2001). Disruption of neurotransmission in *Drosophila* mushroom body blocks retrieval but not acquisition of memory.. Nature.

[36] McGuire SE, Le PT, Davis RL (2001). The role of *Drosophila* mushroom body signaling in olfactory memory.. Science.

[37] Manoli DS, Foss M, Villella A, Taylor BJ, Hall JC (2005). Male-specific fruitless specifies the neural substrates of *Drosophila* courtship behaviour.. Nature.

[38] Stockinger P, Kvitsiani D, Rotkopf S, Tirian L, Dickson BJ (2005). Neural circuitry that governs *Drosophila* male courtship behavior.. Cell.

[39] McCrea DA, Rybak IA (2008). Organization of mammalian locomotor rhythm and pattern generation.. Brain Res Rev.

[40] Ito K, Awano W, Suzuki K, Hiromi Y, Yamamoto D (1997). The *Drosophila* mushroom body is a quadruple structure of clonal units each of which contains a virtually identical set of neurones and glial cells.. Development.

[41] Salvaterra PM, Kitamoto T (2001). *Drosophila* cholinergic neurons and processes visualized with Gal4/UAS-GFP.. Gene Expression Patterns.

[42] Sugimura K, Yamamoto M, Niwa R, Satoh D, Goto S (2003). Distinct developmental modes and lesion-induced reactions of dendrites of two classes of *Drosophila* sensory neurons.. J Neuroscience.

[43] Schuster CM, Davis GW, Fetter RD, Goodman CS (1996). Genetic dissection of structural and functional components of synaptic plasticity. I. Fasciclin II controls synaptic stabilization and growth.. Neuron.

[44] Sanyal S (2009). Genomic mapping and expression patterns of C380, OK6, and D42 enhancer trap lines in the larval nervous system of *Drosophila*.. Gene Expression Patterns.

[45] Fei H, Chow DM, Chen A, Romero-Calderón R, Ong WS (2010). Mutation of the *Drosophila* vesicular GABA transporter disrupts visual figure detection.. J Exp Biol.

[46] Nose A, Umeda T, Takeichi M (1997). Neuromuscular target recognition by a homophilic interaction of connectin cell adhesion molecules in *Drosophila*.. Development.

